# Tumor Associated Macrophage × Cancer Cell Hybrids May Acquire Cancer Stem Cell Properties in Breast Cancer

**DOI:** 10.1371/journal.pone.0041942

**Published:** 2012-07-25

**Authors:** Jingxian Ding, Wei Jin, Canming Chen, Zhiming Shao, Jiong Wu

**Affiliations:** 1 Department of Breast Surgery, Breast Cancer Institute, Fudan University Shanghai Cancer Center, Shanghai, China; 2 Department of Oncology, Shanghai Medical College, Fudan University, Shanghai, China; 3 Fudan University Cancer Institute Research Center, Shanghai, China; University of Medicine and Dentistry of New Jersey, United States of America

## Abstract

Breast cancer is one of the most frequently diagnosed cancers among women, and metastasis makes it lethal. Tumor-associated macrophages (TAMs) that acquire an alternatively activated macrophage (M2) phenotype may promote metastasis. However, the underlying mechanisms are still elusive. Here, we examined how TAMs interact with breast cancer cells to promote metastasis. Immunohistochemistry was used to examine the expression of the M2-specific antigen CD163 in paraffin-embedded mammary carcinoma blocks to explore fusion events in breast cancer patients. U937 cells were used as a substitute for human monocytes, and these cells differentiated into M2 macrophages following phorbol 12-myristate 13-acetate (PMA) and M-CSF stimulation. M2 macrophages and the breast cancer cell lines MCF-7 and MDA-MB-231 fused in the presence of 50% polyethylene glycol. Hybrids were isolated by fluorescence-activated cell sorting, and the relevant cell biological properties were compared with their parental counterparts. Breast cancer stem cell (BCSC)-related markers were quantified by immunofluorescence staining, RT-PCR, quantitative RT-PCR and/or western blotting. The tumor-initiating and metastatic capacities of the hybrids and their parental counterparts were assessed in NOD/SCID mice. We found that the CD163 expression rate in breast cancer tissues varied significantly and correlated with estrogen receptor status (*p*<0.05). The fusion efficiency of either breast cancer cell line with M2 macrophages ranged from 1.81 to 6.47% in the presence of PEG, and no significant difference was observed between the breast cancer cell lines used (*p*>0.05). Characterization of the fusion hybrids revealed a more aggressive phenotype, including increased migration, invasion and tumorigenicity, but reduced proliferative ability, compared with the parental lines. The hybrids also gained a CD44^+^CD24^−/low^ phenotype and over-expressed epithelial-mesenchymal transition-associated genes. These results indicate that TAMs may promote breast cancer metastasis through cell fusion, and the hybrids may gain a BCSC phenotype.

## Introduction

Breast cancer is one of the most frequently diagnosed cancers in women and is the leading cause of cancer-related death among women worldwide, including China. In the United States, it has been estimated that approximately 12% of women will develop breast cancer at some point in their lives [1], [2]. The vast majority of breast cancer-related deaths are due to metastasis, and the lung, liver, bone and brain are the most prevalent sites [3]. Metastasis itself is a well-orchestrated multi-step process. It includes tumor cell invasion of the basement membrane, intravasation into the vascular or lymphatic system, survival in the blood circulation or lymph nodes, attachment to the blood vessel wall and extravasation to the target organ, followed by subsequent colonization and aggressive growth to form a macrometastasis under a favorable microenvironment [4].

The metastatic process is similar to epithelial-mesenchymal transition (EMT), a developmental process during which cells acquire an amoeboid-like phenotype, become motile and disseminate [5]. Most of the current findings support the idea that EMT is the key mechanism by which tumor cells gain invasive and metastatic ability because EMT enables the separation of individual cells from the primary tumor mass [6]. However, the exact mechanism of EMT remains controversial. The hallmarks of EMT include loss of the epithelial molecule E-cadherin and gain of mesenchymal markers, such as N-cadherin and vimentin. The transcription factors Snail1, Snail2 and Twist are key inducers of EMT in cancer cells by repressing E-cadherin expression. By undergoing EMT, cancer cells readily gain access to microvessels [4], [6]. However, the exact mechanisms of cancer metastasis are still unknown. The century-old theory of cancer cell fusion with macrophages may explain the initiation of metastasis [7]. Cell fusion is a process in which two or more cells become one by merging their plasma membranes and rearranging their nuclear contents. The progeny of cell fusion are known as hybrids [8]. The best-known hybrids are hybridomas, which are made by fusing myeloma cells with lymphocytes to produce monoclonal antibodies [9]. Cell fusion is a fundamental process that occurs in both health and disease [10]. The fusion theory of cancer was first proposed by German pathologist Otto Aichel in 1911. While viewing cancer biopsies under the microscope, Aichel observed that white blood cells attacked tumor cells. He proposed that cancer cells and white blood cells might fuse, resulting in the greater number of chromosomes, which is common in cancer cells, and confer a malignant cell with the ability to move through the bloodstream, which is a phenotypic trait of macrophages [11], [12]. Macrophage infiltration is not uncommon in breast cancers. These infiltrative macrophages are called tumor-associated macrophages (TAMs), and in the majority of cases, TAMs enhance tumor progression to malignancy [13]. TAMs may also potentiate the seeding and establishment of metastatic cells [14].

**Table 1:** Clinicopathological characteristics of 89 subjects evaluated for CD163 expression subtypes.

		DCIS	IDC	
			LN(−)	LN(+)
Premenopausal	ER+++	10	8	4
	ER−	11	5	5
Postmenopausal	ER+++	10	6	6
	ER−	12	6	6

Note: 10 normal breast tissue samples obtained from reduction mammoplasty were used as the control. DCIS: Ductal carcinoma in situ. IDC: Invasive ductal carcinoma. LN(−): No lymph node involved. LN(+): Lymph nodes involved. ER+++: >70% of the cells express the estrogen receptor (ER); ER−: No cells express the ER.

Macrophages originate from the mononuclear phagocytic lineage and their polarization is dependent on cytokines in the microenvironment. Classical activation (M1) is triggered by T helper 1 (Th1) cytokines, such as interferon-γ, bacterial lipopolysaccharide (LPS) and TNF-α, while alternative activation (M2) is induced by T helper 2 (Th2) cytokines, such as IL-4, IL-13 and macrophage-colony stimulating factor (M-CSF) [15], [16]. M-CSF is a hematopoietic growth factor that is involved in the proliferation, differentiation and survival of monocytes, macrophages and bone marrow progenitor cells [17]. Notably, TAMs normally share properties of M2 macrophages and promote cancer progression [13], [18], [19].

In the middle of the last decade, John Pawelek noted that heterotypic cell fusion was likely responsible for the change in cancer-cell phenotype and function leading to cancer metastasis [20]. When two cells fuse, their daughter cells share the genetic and functional characteristics of both parent cells. At least three approaches have been developed for fusion-induced reprogramming: electro-fusion, polyethylene glycol (PEG)-induced fusion and Sendai virus-induced fusion [21]. Moreover, different cell types can fuse spontaneously when co-cultured [22], [23]. Of note, numerous tumor cells are fusogenic, and promiscuous fusion between tumor cells or between tumorigenic cells and other cells can endow hybrids with new properties [24]. TAMs also have a high fusogenic potential, which might have an important function in tumor cell fusion events [8]. It is tempting to speculate that stem cells, attracted by mutated cells in a highly fusogenic environment, might themselves become partners in cell–cell fusion events, which might lead to genetic reprogramming and the generation of cancer stem cells (CSCs) [24], [25]. CSCs are a minor subpopulation of cancerous cells that are defined by their ability to self-renew and differentiate to give rise to tumors and the heterogeneous cells within the tumor [26]. The objective of the present study was to examine whether the fusion of TAMs and breast cancer cells results in the genetic reprogramming and generation of CD44^+^CD24^−/low^ breast cancer stem cells (BCSCs), which may contribute to metastasis and relapse [27].

**Figure 1:**
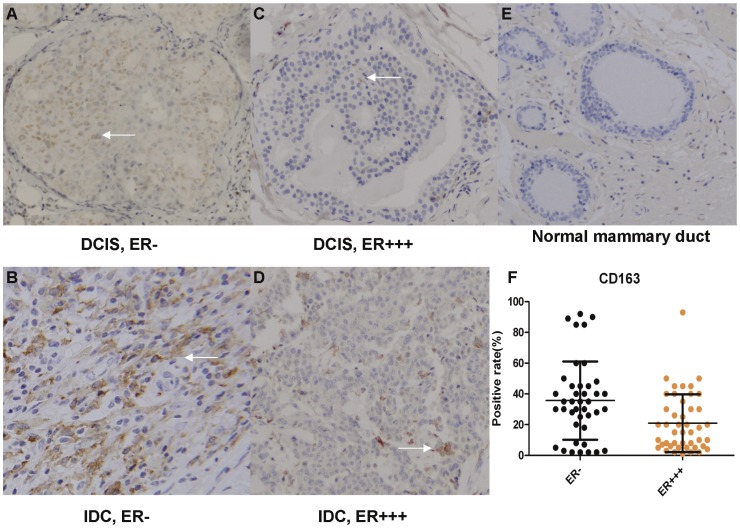
Positive rate of CD163 among breast cancer tissues. Immunohistochemical staining was performed for the M2 marker CD163 (brown) in various human breast cancer tissues. Normal mammary ducts were shown as control. Nuclei were counterstained with hematoxylin (blue) (×100). F: The distribution of CD163-positive rate in breast cancer cells according to ER status. Note: DCIS (ductal carcinoma in situ), IDC (invasive ductal carcinoma), ER (estrogen receptor).

**Figure 2:**
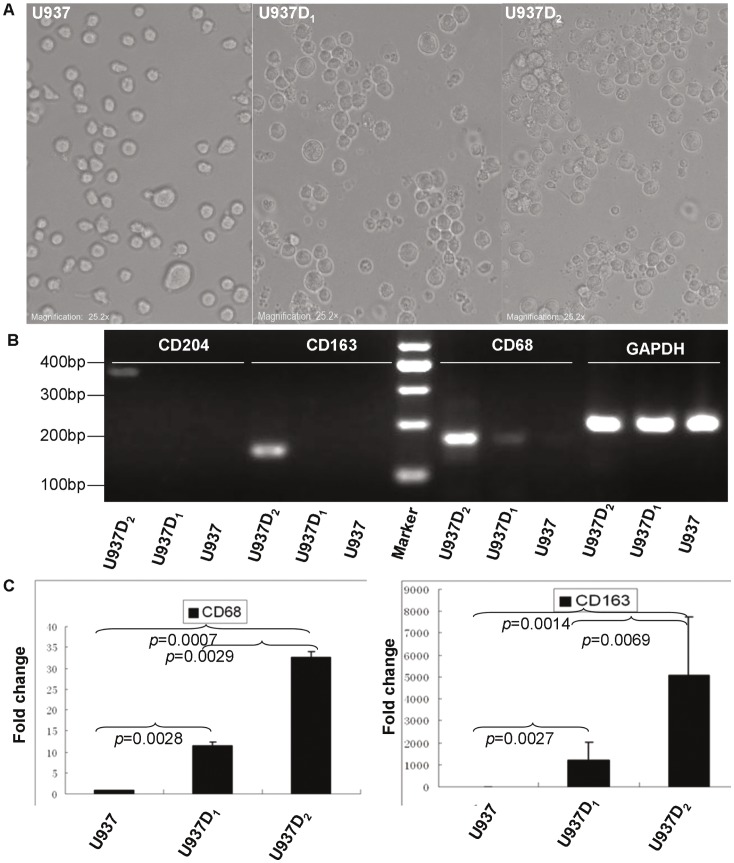
Characterization of U937-derived macrophages. A: Representative images of U937 cells following stepwise differentiation with PMA and M-CSF: U937 (left); U937D_1_ (middle) and U937D_2_ (right) (×200). B, C: The differential expression of macrophage-associated genes among U937, U937D_1_ and U937D_2_ cells detected by RT-PCR (B) and quantitative RT-PCR (C). GAPDH was used as the internal control.

## Materials and Methods

### Ethics Statement

The study involving human participants was approved by the Ethical Committee of Fudan University Shanghai Cancer Center. Written informed consent was obtained from the patients before the enrollment. All animal protocols were approved by the animal care committee of Shanghai Institutes for Biological Sciences of the Chinese Academy of Sciences and Fudan University Shanghai Cancer Center and performed under veterinary supervision. Mice were maintained in laminar flow rooms under constant temperature and humidity.

**Figure 3:**
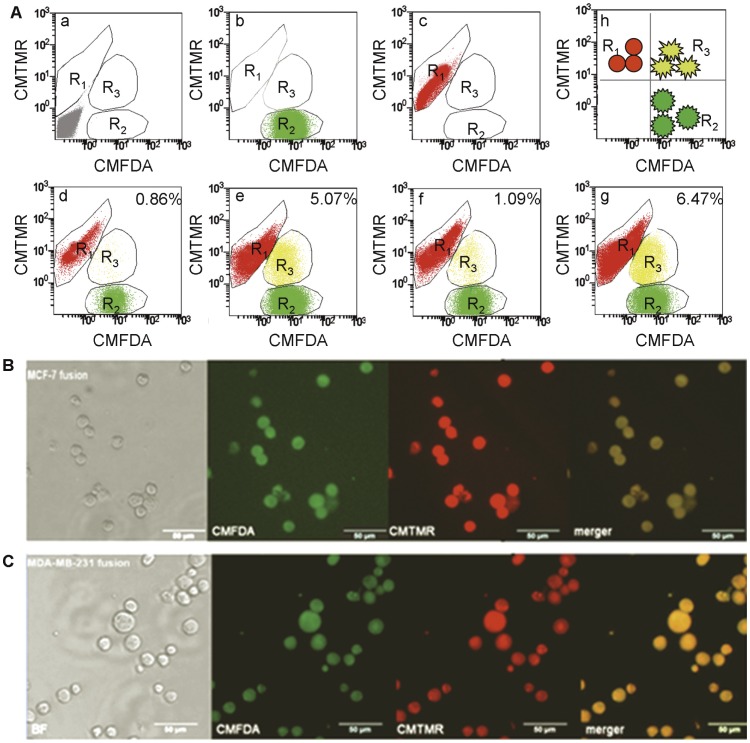
Quantification, isolation and confirmation of fusion hybrids by FACS and fluorescence microscopy. A: Typical FACS dot plots. Unstained mixtures (a), breast cancer cells (b), U937D_2_ cells (c), MCF-7×U937D_2_ without PEG (d), MCF-7×U937D_2_ with PEG (e), MDA-MB-231×U937D_2_ without PEG (f), MDA-MB-231×U937D_2_ with PEG (g), and a schematic of the unfused cells and the hybrids (h). B, C: The purity of the isolated hybrids was almost 100% in both MCF-7 (B) and MDA-MB-231 (C) cell lines, though some cell debris was detected (×200).

**Table 2:** Range and means of fusion efficiency obtained by fusing different breast tumor cell types with U937D_2_ cells.

	MCF-7×U937D_2_	MDA-MB-231×U937D_2_
Mean percentages of fusion efficiency (mean ± SD)	3.50±1.77	4.06±2.07
Range of double positive events (%)	1.81–5.34	1.96–6.47

No significant difference was observed (*p*>0.05).

### Immunohistochemistry

Paraffin-embedded blocks of human breast cancer tissue were obtained from the Breast Malignancy Database established by the Department of Breast Surgery, Fudan University Shanghai Cancer Center, Shanghai, China. All of the enrolled patients have full-detailed clinicopathological information and follow-up results. Written informed consent was obtained from the patients before enrollment. We are authorized to use the tissues for research only and have reported the database information previously [28]. For immunohistochemical analysis, the paraffin-embedded blocks were cut into 5 µm serial sections, and following the confirmation of breast cancer diagnosis by HE staining, immunohistochemistry was performed following standard procedures. CD163 antibody (clone 10D6, Novocastra, England) used for immunostaining was titered to find the optimal concentration (1∶100). Sections were counterstained with hematoxylin for the identification of nuclei. Detection was performed using the DAKO Envision system. Images were captured under a microscope with a CCD camera.

**Figure 4:**
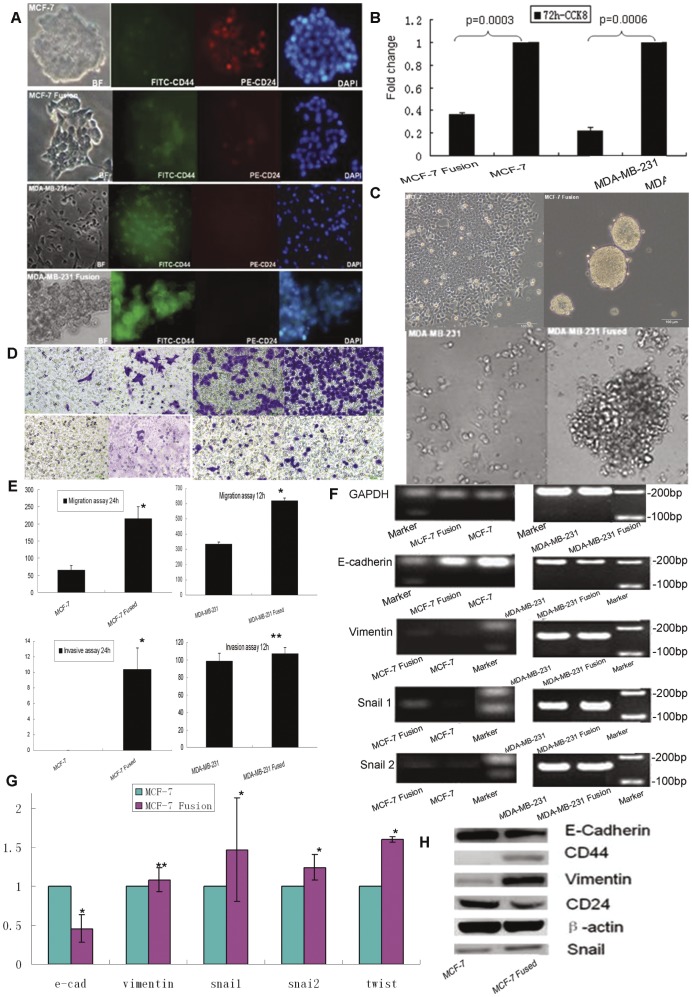
The expression profiles and biological properties of the hybrids and their parental counterparts. Various characteristics were examined in the hybrids and their parental counterparts. A: Analysis of CD44 and CD24 expression by immunofluorescence staining (×200), B: Proliferation, as determined by CCK-8 assay, and C: mammosphere formation ability (×100). D/E: Transwell assays were used to examine the migrative and invasive ability of hybrids and parent cells (×200). F–H: Gene expression of E-cadherin, vimentin, snai1, snai2 and twist was analyzed by RT-PCR (F), quantitative RT-PCR (G) and western blotting (H). (*p<0.05, **p>0.05).

**Figure 5:**
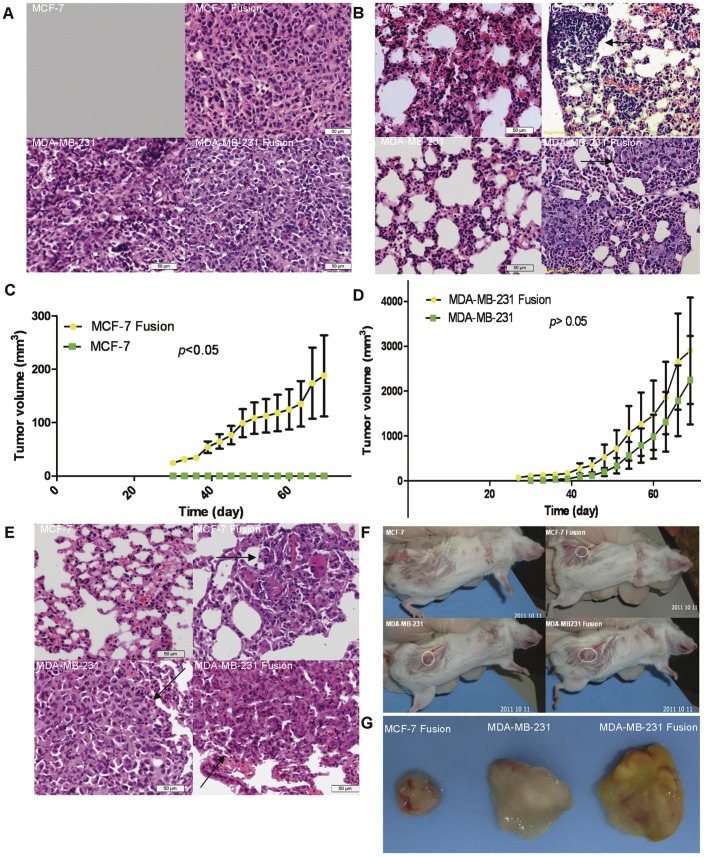
Tumorigenicity of hybrid xenografts in NOD/SCID mice. Tumor formation and growth were monitored in NOD/SCID mice orthotopically injected with hybrids or parental breast cancer cell lines. Lung metastasis was also examined in NOD/SCID mice after tail-vein injection with hybrids or parent cells. A: The orthotopic tumors. B: The orthotopic tumors metastasizing to the lungs. C, D: Tumor growth curves of MCF-7 and MDA-MB-231 hybrids; data are shown as the mean ± standard error of 3 mice. E: Lung metastasis after tail vein injection. F: Representative NOD/SCID mice tumors after orthotopic injection of 5×10^5^ cells. G: A representative images of tumors excised from each group as indicated (×200).

### Cell Culture and Preparation of M2 Macrophages

Breast cancer cell lines MDA-MB-231 and MCF-7 were originally obtained from the ATCC (American Type Culture Collection, Manassas, VA, USA); the human promonocytic cell line U937 originating from the ATCC was kindly provided by Prof. Ma (Key Laboratory of Molecular Medicine, Ministry of Education, Shanghai Medical College, Fudan University). All cells were maintained in RPMI 1640 supplemented with 10% fetal bovine serum (FBS) and 100 units/ml penicillin/streptomycin (GIBCO, Invitrogen, Carlsbad, CA, USA) at 37°C in a humidified atmosphere of 5% CO2. To induce U937 differentiation into macrophage-like cells (U937D_1_), PMA (Sigma-Aldrich, St. Louis, MO, USA) dissolved in DMSO (Sigma-Aldrich) was added to 5×10^5^ cells/ml at a final concentration of 10 ng/ml for 72 hours. U937D_1_ cells were skewed toward M2 macrophages (U937D_2_) in the presence of 10 ng/ml M-CSF for an additional 7 days.

**Figure 6:**
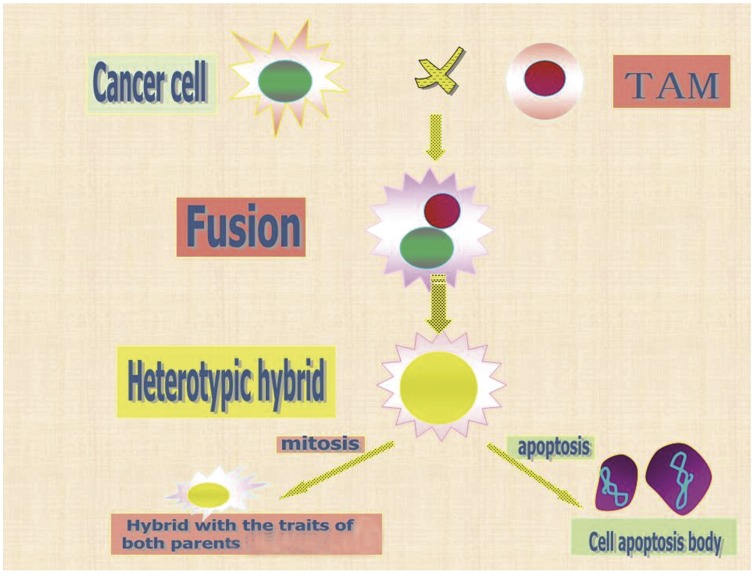
Diagram of the cell fusion process and proceeding events. Monocytes, chemoattracted to tumor sites, were induced to become tumor-associated macrophages (TAMs). The latter then merged with cancer cells to form fusion hybrids. After successful mitosis, the progenies of the hybrids acquired traits of both of their parents; otherwise, the fusion hybrids underwent apoptosis.

### Cell Tracker Dye Staining

MCF-7 and MDA-MB-231 breast cancer cell lines were stained with 1.25 µg/ml CMFDA (green, Molecular Probes, Eugene, OR, USA), and U937D_2_ cells were stained with 2.5 µg/ml CMTMR (red, Molecular Probes) for 30 min at 37°C. Each suspension was washed thrice with PBS (Gibco); the pellets were collected and resuspended in one staining volume of growth media and then incubated for 60 min in the incubator, shielded from light. The cells were then examined under a fluorescence microscope to confirm staining, with non-stained cells used as the control. Of note, the dyes used can pass freely through membranes into the cytosol. However, both dyes undergo a glutathione 5-transferase mediated reaction in the cytosol that renders the dyes membrane impermeable [29].

**Figure 7:**
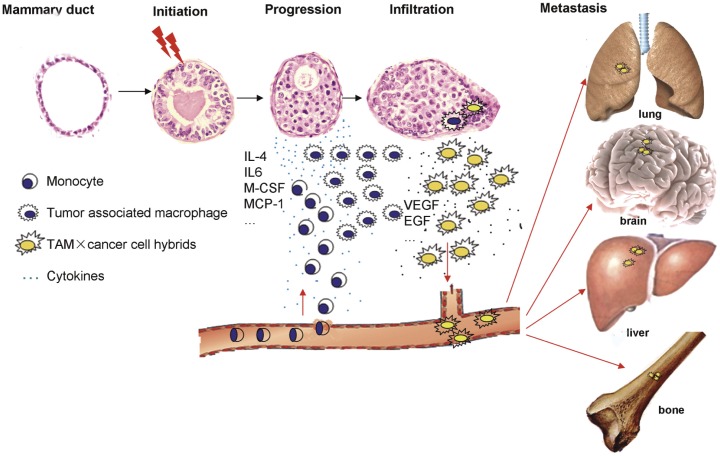
Schematic representation of the probable development of breast cancer. Normal mammary ducts suffering from two-hits undergo malignant transformation. The transformed cells secrete chemotactic factors (e.g., IL4, IL6, M-CSF, and MCP-1), which chemoattract monocytes and induce their differentiation. The differentiated monocytes become fusogenic and fuse with transformed cells, resulting in hybrids harboring traits of both parent cells. These hybrids acquire BCSC properties after nuclear reprogramming. Finally, the hybrids detach and metastasize to distant organs.

### PEG-mediated Cell Fusion

Hybrids were generated by fusing U937D_2_ with each breast cancer cell line using 50% PEG 1450 (Sigma-Aldrich), as previously reported [30]. Briefly, pre-stained or unstained U937D_2_ cells were mixed with breast cancer cells at a ratio of 1∶1, followed by centrifugation at 200 g for 5 min. The supernatant was skimmed off to prevent PEG dilution. The cell pellets were dissociated, and 1 ml of pre-warmed 50% PEG solution was added drop-wise for 1 min with gentle mixing, followed by 1 min of incubation at 37°C. Next, 1, 3 and 6 ml of RPMI 1640 were added, each over 30 s, followed by mixing and 1 min of incubation. Finally, the cell suspensions were centrifuged at 200 g for 5 min, and the cell pellets were resuspended in complete RPMI 1640 medium and cultured overnight. The mixture of U937D_2_ cells with MCF-7 or MDA-MB-231 cells in the absence of PEG was used as a no-fusion control.

### Fluorescence-activated Cell Sorting (FACS)

Cells were trypsinized using 0.25% EDTA-Trypsin (Gibco), washed three times, and resuspended in PBS containing 0.5% FBS. Cell clumps were removed by passing cell suspensions through 40 µm Cell Strainers (BD Biosciences, Bedford, MA, USA). Flow cytometric analysis of the cell tracker dyes was used to identify successful fusions. Briefly, a plot of FL1 (green fluorescence) and FL2 (red fluorescence) was made to ensure that each fusion partner had a suitable fluorescent magnitude. Excitation was provided by an 80 MW Argon laser tuned to the wavelength of 488 nm. The emission of CMFDA and CMTMR was detected by FL1 and FL2, respectively. The hybrids, which appeared as a dual-fluorescing population, were isolated by FACS (Beckman Coulter Inc., USA). The mixtures of U937D_2_ cells with MCF-7 or MDA-MB-231 cells without PEG treatment were used as controls. The unstained mixtures, pre-stained U937D_2_ cells and breast cancer cells were used to set the gates.

### Immunofluorescence Staining

Cells were washed three times with PBS, fixed with ice-cold methanol for 15 min and permeabilized with 0.3% Triton X-100-PBS for 5 min prior to blocking with 10% goat serum-PBS for 1 hour at room temperature (RT). The cells were then incubated with FITC-conjugated CD44 and PE-conjugated CD24 antibodies (BD Biosciences) for 2 hours at RT. Cell nuclei were visualized with DAPI (Sigma-Aldrich). The cells were rinsed with PBS and air dried before imaging under an inverted fluorescent microscope equipped with a CCD camera.

### Cell Proliferation Assay

Cell proliferation was assessed using CCK-8 (Dojindo Laboratories, Kumamoto, Japan). Briefly, the hybrids and their parental counterparts were plated in triplicate at 5,000 cells/well in a 96-well plate. After incubation for the indicated time, 10 µl/well of CCK-8 was added, and the cells were incubated at 37°C for 2 hours. Optical density was measured at 460 nm using a microplate reader. Three readings were obtained, with wells without cells used as the blank control.

### Mammosphere Formation Assay

Mammosphere formation assays were performed as previously described by Charafe-Jauffret with a slight modification [31]. Briefly, 2×10^5^ cells/well were grown in six-well Ultra Low Attachment plates (Costar, Corning, NY, USA) in 2 ml serum-free RPMI 1640 medium. After 7 days of culture, the number and size of mammospheres for each well were blindly evaluated under an inverted microscope by two independent observers.

### Transwell Assay for Migration and Invasion

Cells were trypsinized using 0.25% EDTA-Trypsin, resuspended in RPMI 1640 containing 0.5% FBS, and then transferred into polyethylene terephthalate membrane Transwell niches (24-well inserts, 8 µm pore-size, Costar, Corning, NY, USA) with or without the Matrigel™ basement membrane matrix (BD Biosciences) at 5×10^4^ cells/well in a volume of 100 µl RPMI1640 medium for invasion or migration assays. The lower chamber was filled with 0.5 ml of complete medium to serve as a chemoattractant. After incubation for 24 hours at 37°C, the cells in the upper chamber were carefully scraped off with swabs, while the cells penetrating the membrane were fixed with ice-cold methanol and stained with 0.1% crystal violet. Following air-drying, the cells were imaged using a microscope with a CCD camera. Five random visual fields (×100) were counted. The invasion or migration ability of the tumor cells was quantified by averaging the number of positively stained cells in each microscopic field.

### Reverse Transcription–PCR Analysis

Total RNA was isolated from 1×10^6^ cells using Trizol (Invitrogen) according to the manufacturer’s directions. One microgram of total RNA was reverse transcribed using PrimeScript® RT-PCR Kit (Takara, Dalian, China) primed with oligo(dT). One microliter of cDNA was subjected to PCR amplification using gene-specific primers. PCR products were resolved on a 2% agarose gel and visualized by ethidium bromide staining. Quantitative PCR was carried out using the SYBR®Green PCR Mix Kit (Takara). Primer sequences are detailed in Table S1. The quantification of gene expression was normalized to the expression of GAPDH.

### Western Blotting Assay

For immunoblot analysis, cell lysates containing 50 µg of total protein were separated by electrophoresis on 10% sodium dodecyl sulfate–polyacrylamide gels and electroblotted onto polyvinylidene difluoride membranes (Millipore, Billerica, MA). After blocking of the unoccupied sites with 5% skim milk-PBS, the membranes were probed with the desired primary antibodies at 4°C overnight, followed by incubation with appropriate HRP-conjugated secondary antibodies, according to manufacturer’s instructions. The primary antibodies were anti-β-actin, anti-Snail, anti-Vimentin, anti-E-cadherin (Proteintech Group, Inc., USA), anti-CD44 and anti-CD24 (NeoMarkers, Fremont, CA, USA). The signal was visualized by ECL Western Blotting Detection Reagents (Pierce) and photographed with ImageQuant LAS 4000 (GE Healthcare, Piscataway NJ, USA). β-actin was used as a loading control.

### Breast Cancer Xenografts

To assess the hybrid tumorigenicity in vivo, 8-week-old female non-obese diabetic/severe combined immunodeficient (NOD/SCID) mice were used. For tumorigenicity studies, 5×10^5^ cells suspended in 100 µl PBS were injected orthotopically into the mammary fat pad according to standard injection procedures. Once tumors were palpable, tumor growth was monitored every other day for 10 weeks. The tumor volume was calculated as (width^2^×length)/2. Tumor growth curves were plotted. The mice were sacrificed by cervical dislocation and necropsied 10 weeks after injection. Tumors and lungs were excised, weighed and processed for HE staining. For the in vivo metastasis assay, 2×10^5^ cells suspended in PBS were injected into the tail veins of mice. To reduce the possibility of lung embolism, cell suspensions were passed through 40 µm Cell Strainers to remove cell clumps. The mice were sacrificed and necropsied after 8 weeks. Lung metastases were observed and imaged following HE staining.

### Statistical Analysis

The quantitative results are represented as the mean ± standard error of at least three independent experiments. Significant differences were determined with Student’s t test using EXCEL or GraphPad Prism 5 Demo (GraphPad Software, San Diego, CA, USA). A *p*<0.05 was considered statistically significant.

## Results

### Expression of the M2 Macrophage Marker CD163 in Breast Cancer

To assess the tumor-associated macrophage and breast cancer cell fusion events in breast cancer patients, we selected 89 paraffin-embedded mammary carcinomas blocks, 45 of which were estrogen receptor (ER) positive. Ten normal breast tissue samples, obtained from reduction mammoplasty, were used as an internal control (Table 1). The mean positive rate of CD163, which has been reported to be a relatively specific marker of M2 macrophages [32], [33], was 21% (ranging from 1% to 93%) and 35% (ranging from 2% to almost 92%) among breast cancer cells in ER+++ and ER- cases, respectively (*p*<0.05). A typical image of each group is shown in Figure 1. Utilizing the Kaplan–Meier Plotter tool [34], we found that CD163 expression was an independent prognosis factor in a large multi-study data set (Figure S1).

### Characterization of U937-derived M2 Macrophages

The human promonocytic U937 cells normally grow in suspension and have a smooth surface; however, exposure to PMA (10 ng/ml, 72 hours), they were induced to differentiate into U937D_1_ cells, as evidenced by a thorny morphology, attachment to the flask surface, and increased expression of the putative macrophage surface antigen CD68 #. U937D_1_ cells were further skewed toward an M2 phenotype, referred to as U937D_2_, by co-culturing with M-CSF (10 ng/ml, 7 days). The U937D_2_ cell phenotype was confirmed by CD163 and CD204 expression, both of which are characteristic of M2 macrophages [33], [35]. No further obvious morphological changes were observed in U937D_2_ cells (Figure 2). U937D_2_ cells, regarded as TAMs, were used for further investigation.

### Percentage of Double-positive Events in MCF-7 and MDA-MB-231 Cells

Each fusion partner was stained with either CMTMR or CMFDA fluorescent dyes. The fusion efficiency represented by double-positive cells was analyzed by FACS analysis. To determine whether the induced fusion efficiency was dependent on the tumor cell line, both MCF-7 and MDA-MB-231 cells were fused with U937D_2_ cells. The mean percentage of fusion efficiency was 3.50% for MCF7 cells, ranging from 1.81 to 5.34%, and 4.06% for MDA-MB-231 cells, ranging from 1.96 to 6.47% (Table 2). The mean percentage of spontaneous double-positive events was approximately 1% for all cell types. No significant differences were observed in double-positive events obtained, though MDA-MB-231 cells showed a higher efficiency (*p*>0.05). The discrepancy of fusion efficiency in our experiment may be partially attributed to our stringent gate used to obtain pure hybrids for further study [36]. A representative FACS plot for each group was applied, and the purity of the isolated hybrids was nearly 100%, as confirmed by fluorescent microscopy (Figure 3).

### MCF-7 and U937D_2_ Hybrids Exhibited a CD44^+^/CD24^−/low^ BCSC Phenotype

Dual-immunofluorescence detection by flow cytometry for CD44 and CD24 was applied to compare the frequency of the CD44^+^/CD24^−/low^ BCSC phenotype between the hybrids and their parental counterparts. MCF-7 and U937D_2_ hybrids exhibited enhanced CD44 expression but decreased CD24 expression compared with parental MCF-7 cells, indicating that the hybrids had acquired a CD44^+^/CD24^−/low^ phenotype. Moreover, the morphology was also changed, as the cells became more fibril-like (Figure S2). Notably, the MCF-7 cell line scarcely exhibits a CD44^+^/CD24^−/low^ phenotype [37]. However, a similar phenomenon was not detected in the MDA-MB-231 and U937D_2_ hybrids, which may be partially due to MDA-MB-231 cells alone having a high percentage of BCSCs [37] (Figure 4A).

### The Fusion Hybrids Acquired Several BCSC Traits

For cell proliferative ability, the growth rate for each group was measured using the CCK-8 assay over a 72 hour period. The hybrid proliferative index was 21.5% and 34.6% of the MCF-7 and MDA-MB-231 parental cell lines, respectively (Figure 4B, *p*<0.05). Thus, the fusion hybrids of breast cancer cells and M2 macrophages were less proliferative.

Next, the mammosphere formation assay was used to test the ability of cells to resist anoikis, which is a key property of BCSCs [38]. Here, we found that the hybrids of breast cancer cells and U937D_2_ formed more spheres of larger size compared to their parents after being plated in non-adherent mammosphere culture conditions for seven days (Figure 4C).

In vitro migrative and invasive abilities of the hybrids were also compared with their parental counterparts using Transwell assays. The migrative and invasive ability of the MCF-7 and U937D_2_ hybrids was enhanced significantly compared with those of MCF-7 cells, while the MDA-MB-231 and U937D_2_ hybrids only demonstrated this phenomenon in the migration assay. No significant differences were detected in the invasive ability of MDA-MB-231 and U937D_2_ hybrids, although the number of the hybrids that penetrated the Matrigel™ basement membrane was also higher than that of the parent cell line (Figure 4D, 4E).

### EMT-associated Genes are Differentially Expressed between the Hybrids and the Parent Lines

To explore whether fusion resulted in an EMT-associated phenotype, the expression of important genes related to EMT was examined. The expression of E-cadherin was reduced, while the expressions of vimentin, snail1, snail2 and twist were increased after MCF-7 cells fusion with U937D_2_ cells, as detected by RT-PCR, quantitative PCR and western blotting (Figure 4F, 4G, and 4H). However, we did not detect the differential expression of those genes between MDA-MB-231 and U937D_2_ hybrids and MDA-MB-231 alone, which may be partially due to the weak expression of E-cadherin and the strong expression of vimentin, snail1 and snail2 in MDA-MB-231 cells.

### Hybrids Exhibited Enhanced Tumorigenicity and Metastatic Ability in NOD/SCID Mice

Tumor-initiating potential was evaluated between hybrids and their corresponding parent lines in NOD/SCID mice. MCF-7 and U937D_2_ hybrids initiated orthotopic tumors in 100% (3/3) of the mice within 10 weeks, with two animals developing lung metastases, as confirmed by HE analysis. In contrast, no tumors or lung metastases were detected in mice injected with the same number of MCF-7 cells alone. For the MDA-MB-231 group, both MDA-MB-231 and U937D_2_ hybrids and MDA-MB-231 cells alone formed orthotopic tumors within 10 weeks. Although the former caused larger tumors in general, no statistical significance was observed (*p*>0.05). Strikingly, lung metastases were detected in all mice injected with MDA-MB-231 and U937D_2_ hybrids, but were not found in the group injected with only MDA-MB-231 cells at the same time point. For the in vivo model of breast cancer metastasis, we first intravenously transplanted cells through the tail vein and then monitored the mice for lung metastases. All recipients had lung metastases of varying severity after 8 weeks, except for the mice injected with MCF-7 cells alone, as confirmed by HE staining. However, the severity varied (Figure 5, Table S2 and Figure S3).

## Discussion

Cancer is a heavy burden on public health, and one of the leading causes of disease-associated death [2]. Though great progress has been made in cancer prevention and treatment, its carcinogenesis is still ambiguous. Metastasis largely underlies the difficulty in successfully treating cancer. Thus, clarifying how metastasis occurs could be crucial for the identification of novel therapeutic targets to improve cancer treatment. EMT aids cell motility, the key prerequisite for tumor cell dissemination. Recent reports have shown that the induction of EMT in immortalized human breast epithelial cells is associated with the acquisition of BCSCs-associated properties, as demonstrated by the increased expression of CD44^+^/CD24^−/low^ cells as well as the ability to form mammosphere colonies in culture [39]. Taken together, the gain of motility and the acquisition of CSCs-associated properties by cancer cells could pave the way to metastasis.

The idea that cell fusion contributes to cancer progression was introduced almost 100 years ago with a proposal that malignancy is a consequence of hybridization between leukocytes and somatic cells, and Melanoma × macrophage hybrids with enhanced metastatic potential [40]. Years later, this idea was expanded to encompass that cell fusion promotes the phenotypic and genotypic diversity of tumors and that the fusion of tumor cells with leukocytes results in metastatic cells [41], [42]. Cell fusion is a part of normal development and tissue homeostasis. The fusion of normal somatic cells is a tightly controlled process that is restricted to only a few cell types in humans and results in terminally differentiated multinuclear cells incapable of proliferation, such as syncytiotrophoblasts, myoblasts, and osteoclasts [10]. Intriguingly, as a fusion partner, tumor cells appear to violate the strict rules of cell fusion. Hybridization between TAMs and breast cancer cells as a mechanism for breast cancer metastasis presents the cancer cell in a different light: such hybrids, with features of both parental lineages, can transfer to blood circulation freely, as illustrated in Figure 6. Although their genetic complement would be random at the very beginning, common traits would emerge based on Darwin’s theory of evolution: survival of the fittest, which could be due to the survival benefits derived from some gene-expression patterns or to the nature of hybridization that controls gene expression in hybrid genomes of different embryonic lineages by unknown mechanisms [10].

The onset of metastasis is still unclear; however, breast cancer metastasis seems to be at least partially due to the acquisition of myeloid-type traits #. In breast cancer, the abundance of infiltrating macrophages has been correlated with poor prognosis, and genes associated with macrophage infiltration are part of a molecular signature that heralds negative prognosis in node-negative, tamoxifen-treated breast carcinomas [43]. Though the expression of M2 macrophage-specific antigen CD163 varied significantly in primary breast cancer, its prevalence has a prognostic impact on both relapse-free survival and overall survival (Figure S1), which could be explained by fusion between macrophages and cancer cells [44]. The fusion theory of cancer states that the gain of metastatic ability is derived from cancer cell fusion with a migratory leukocyte. This resultant hybrid adopts the leukocyte’s natural ability to migrate throughout the body while continuing to grow in the uncontrolled manner of the original cancer. This model is also known as the “wolf in sheep’s clothing” model and explains how hybrids evade immune supervision. Additionally, periodically refreshing the genome with bone marrow-derived cells may contribute to telomeric maintenance, which is essential for the survival of tumor cells and may be a characteristic of CSCs [45].

In a very recent article, Rappa G and colleagues reported that a spontaneous in vitro formation of heterotypic hybrids between human bone marrow-derived multipotent stromal cells (MSCs) and breast carcinoma cell lines (MDA-MB-231 and MA11) exhibits a more aggressive behavior [46]. Here, we used the U937 cell line as a substitute for primary human monocytes. Although the use of primary cells would allow stronger conclusions, the enrichment of primary human monocytes involves many ethical issues. We have demonstrated that the tumorigenicity and invasiveness of the fusion hybrids increased significantly in the weakly tumorigenic MCF-7 breast cancer cell line without estrogen implantation. However, the proliferative ability of the fused cells is weaker than their parental counterpart in vitro, which may be interpreted that the hybrids may undergo programmed cell death if reprogramming fails after fusion, as illustrated in Figure 6. The essence of cancer stem cells, which are in a quiescent state but can rapidly expand and differentiate in response to environmental cues, may be also possible [47].

To our knowledge, this is the first report demonstrating direct experimental evidence that fusion of TAMs and breast cancer cells is a possible source of BCSCs, which may be the driver of metastasis and relapse. Based on this finding and previous reports, we graphically depicted the probable development of breast cancer in Figure 7. However, not all patients with breast cancer can educate monocytes to TAMs and form hybrids; therefore, the prognosis varies significantly even with early breast cancer. Thus, some patients may evade aggressive treatment for early disease after surgical intervention.

In summary, TAMs play important role in breast cancer progression, and further studies are essential to elucidate how circulating monocytes are recruited to the tumor microenvironment and differentiate into TAMs. New strategies for tumor targeting and targeted chemotherapy could emerge from a better understanding of hybrid genetics and biology. Consequently, preventative treatment based on the suppression of tumor-cell fusion might be possible.

## Supporting Information

Figure S1 **CD163 gene expression is predictive of recurrence-free survival in breast cancer.** A: RFS (relapse-free survival) analysis of CD163 gene expression in 2898 patients with different clinicopathological characteristics. B: OS (overall survival) analysis of CD163 gene expression in 791 patients.
(TIF)

Figure S2 **The morphological characteristics of MCF-7 and its fusion hybrid.** MCF-7 cells form clusters or domes (left), while the hybrids are more stretched out looking (fibril-like) and have less cell-cell contacts, look quite similar to MDA-MB-231 cells (right). x100
(TIF)

Figure S3 **Representative lung metastasis through mouse tail vein injection.** The severity of lung metastasis was examined by HE. Pictures were processed by photoshop. x40
(TIF)

Table S1 **Characteristics of PCR Primer Sets and Products.**
(DOC)

Table S2 **Tumorigenicity of the hybrids was enhanced in NOD/SCID mice.**
(DOC)
