# Expression of Concern: Tumor Associated Macrophage × Cancer Cell Hybrids May Acquire Cancer Stem Cell Properties in Breast Cancer

**DOI:** 10.1371/journal.pone.0342526

**Published:** 2026-02-10

**Authors:** The PLOS One Editors

Following the publication of this article [1], concerns were raised regarding results presented in Figs 2, 4, and 7, and the methods. Specifically:

- The upper region of Fig 1B appears to overlap with Fig 7B in a later article [2].
- In Fig 4F:
  - Several panels appear to contain vertical discontinuities between the right lane and the remainder of the panel.
  - The band sizes in the left Snail 1 panels do not appear to match the corresponding product size.
  - The MDA-MB-231 Fusion lane in the right GAPDH panel appears similar to the Marker lane in the right Snail 2 panel.
  - The Marker lanes in the right E-cadherin and Vimentin panels appear similar.
- Student’s t-test may not be appropriate for comparing the three variables in Fig 2C.
- The Ethics Statement does not report an ethics approval number.
- The Materials and Methods do not describe the humane endpoints applied.

The first author stated that Fig 1B in [1] is incorrect, and provided replacement images from the original experiments for Figs 1A-E and a corrected Fig 1. The underlying images for the updated Fig 1 are provided in S1 File, and the individual-level underlying data for Fig 1F are provided in S2 File. Underlying images for the remainder of the 89 samples were not provided on editorial request.

The first author also stated that, in Fig 4F, the size markers were spliced into the gel images for visualization purposes. The original, uncropped gel images for Fig 4F were not provided upon editorial request and therefore these concerns cannot be fully resolved. The first author provided some underlying data for Figs 4E and G (S4 File). They also stated that the product sizes of all lanes in the right-hand panel of the GAPDH samples and the size Marker lanes of both Snail 1 panels in Fig 4F are incorrect. The first author provided an updated Fig 4 with corrected product size labels (S5 File).

A statistical reviewer reanalyzed the underlying data for Fig 2C provided by the first author in S3 File. Using ANOVA, with p values adjusted with Bonferroni’s method for pairwise group comparison, they stated that the p-values remain significant.

The first author stated that the animal experiments in [1] were outsourced to an external company, and that they are unable to provide any approval documentation or further information regarding the animal experiments carried out in this study.

The first author also stated that the remainder of the underlying data for [1] are available upon request.

The *PLOS One* Editors issue this Expression of Concern to notify readers of the above concerns and to relay the data provided by the first author.

**Fig 1:**
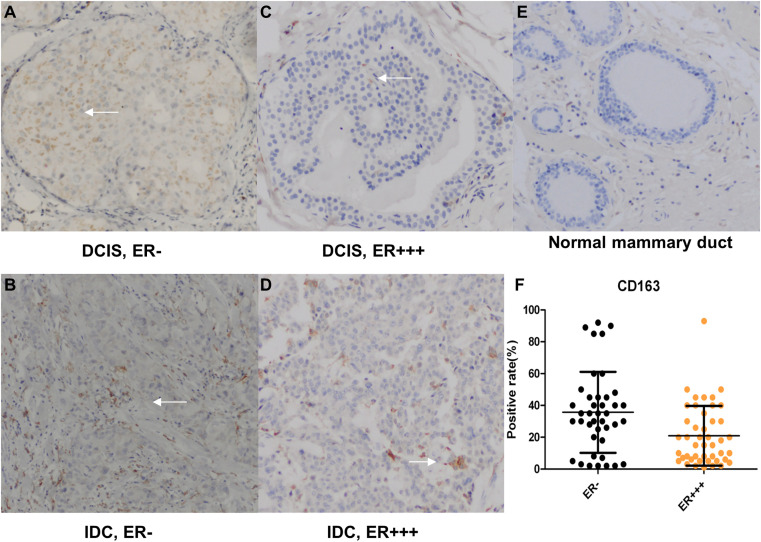
Positive rate of CD163 among breast cancer tissues. Immunohistochemical staining was performed for the M2 marker CD163 (brown) in various human breast cancer tissues. Normal mammary ducts were shown as control. Nuclei were counterstained with hematoxylin (blue) (×100). F: The distribution of CD163-positive rate in breast cancer cells according to ER status. Note: DCIS (ductal carcinoma in situ), IDC (invasive ductal carcinoma), ER (estrogen receptor).

## Supporting information

S1 File Underlying images in support of Figs 1A-E. (ZIP)

S2 File Original quantitative data underlying Fig 1F. (PZF)

S3 File Original quantitative data underlying Fig 2C. (XLS)

S4 File Original quantitative data underlying Fig 4G and partial original quantitative data underlying Fig 4E. (ZIP)

S5 File Figure 4 with updated product size labelling. (TIF)
