# Supplementary figures and images for: Tumor Associated Macrophage × Cancer Cell Hybrids May Acquire Cancer Stem Cell Properties in Breast Cancer
Source: PLoS One. 2012 Jul 25;7(7):e41942. doi: 10.1371/journal.pone.0041942 (PMC3405038; doi:10.1371/journal.pone.0041942)

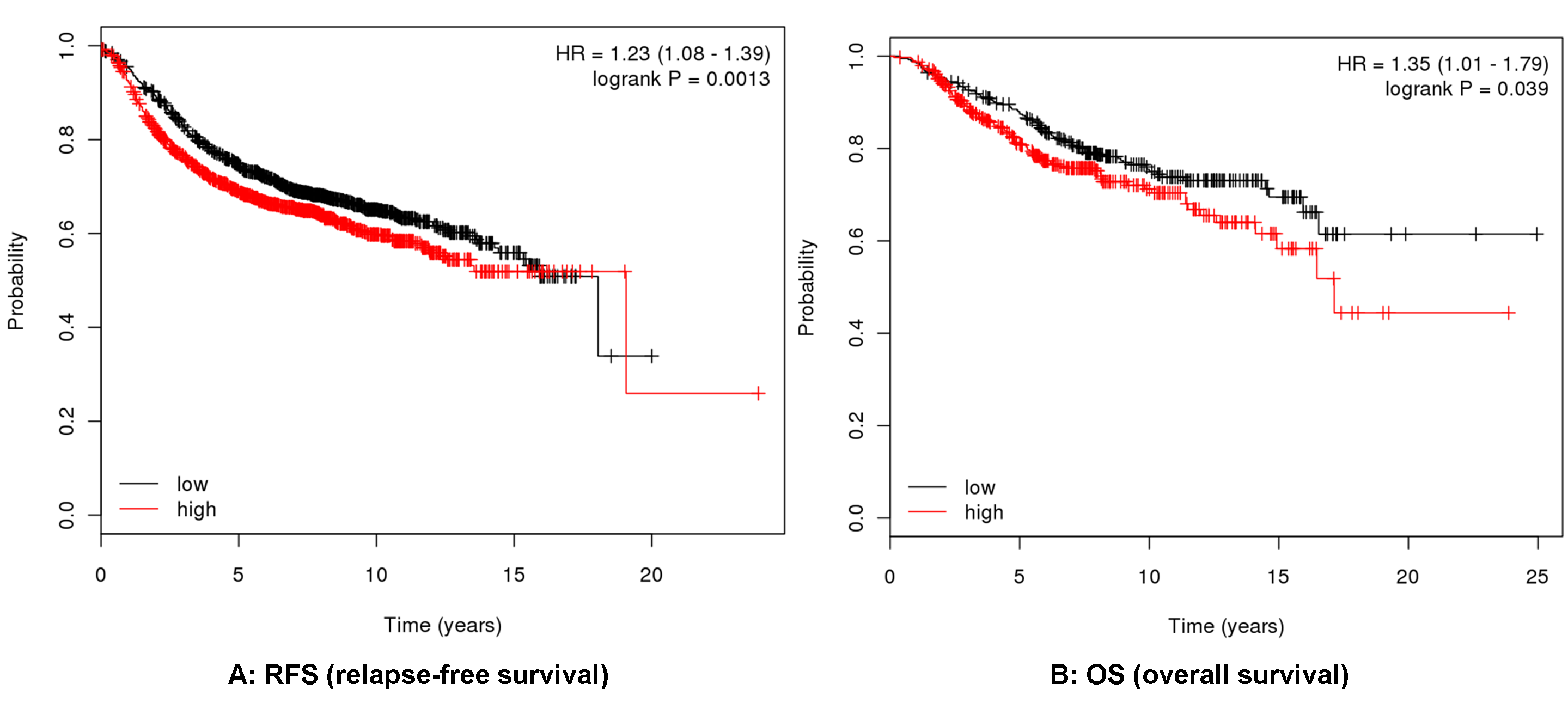

Supplement: Figure S1 — CD163 gene expression is predictive of recurrence-free survival in breast cancer. A: RFS (relapse-free survival) analysis of CD163 gene expression in 2898 patients with different clinicopathological characteristics. B: OS (overall survival) analysis of CD163 gene expression in 791 patients. (TIF) [file pone.0041942.s001.tif]

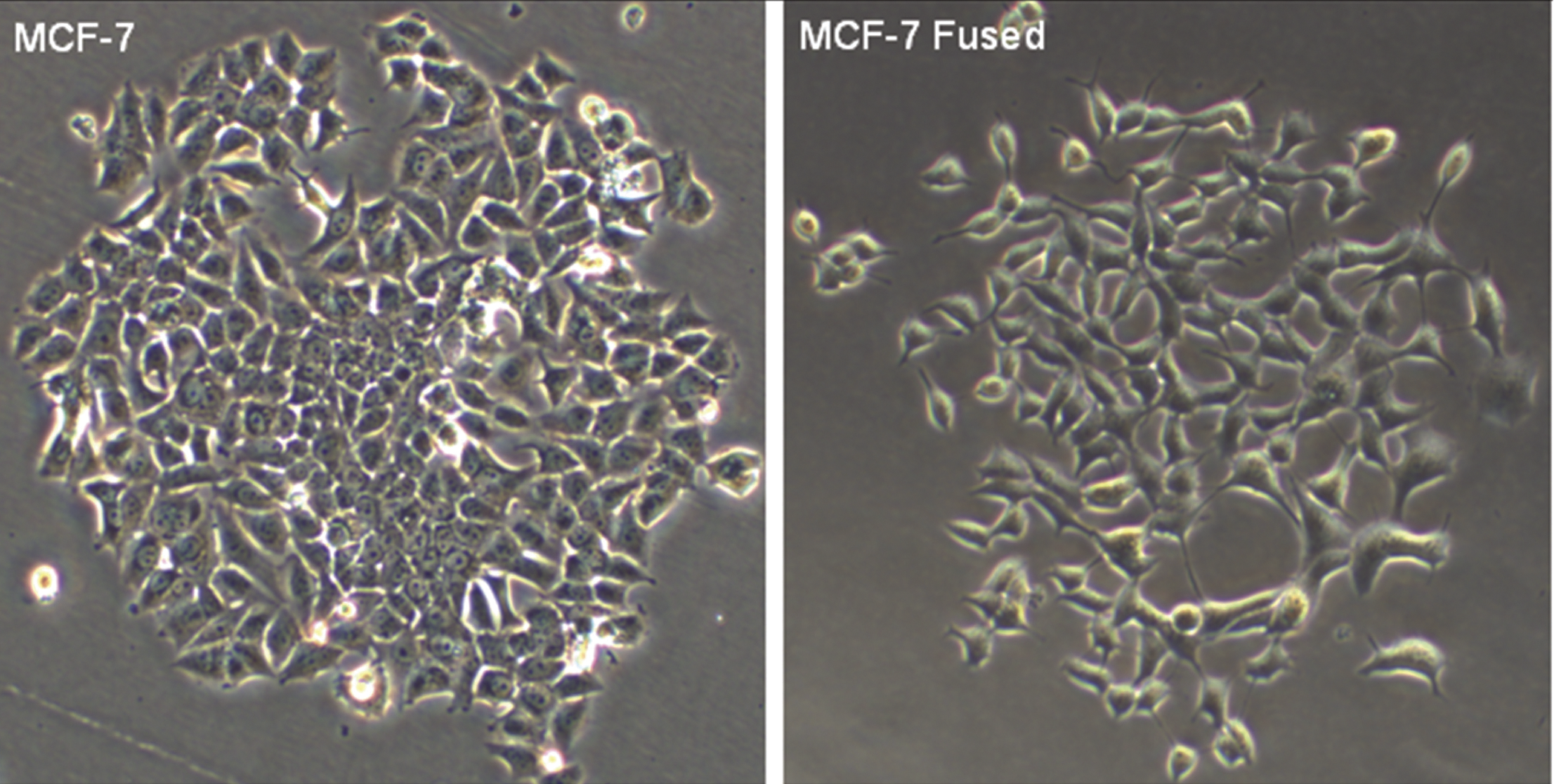

Supplement: Figure S2 — The morphological characteristics of MCF-7 and its fusion hybrid. MCF-7 cells form clusters or domes (left), while the hybrids are more stretched out looking (fibril-like) and have less cell-cell contacts, look quite similar to MDA-MB-231 cells (right). x100 (TIF) [file pone.0041942.s002.tif]

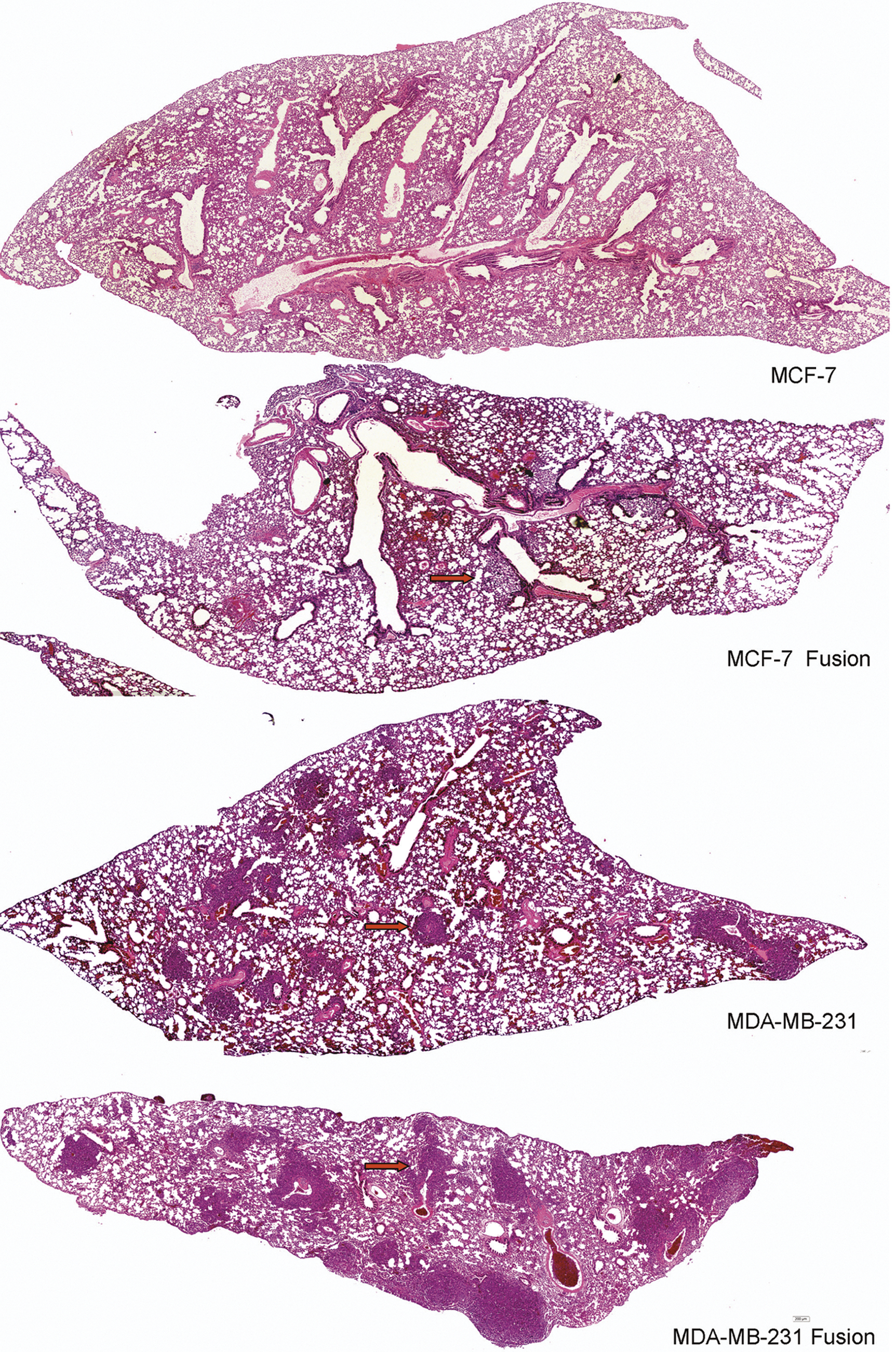

Supplement: Figure S3 — Representative lung metastasis through mouse tail vein injection. The severity of lung metastasis was examined by HE. Pictures were processed by photoshop. x40 (TIF) [file pone.0041942.s003.tif]
