# Supplementary material for: Tumor Associated Macrophage × Cancer Cell Hybrids May Acquire Cancer Stem Cell Properties in Breast Cancer
Source: PLoS One. 2012 Jul 25;7(7):e41942. doi: 10.1371/journal.pone.0041942 (PMC3405038; doi:10.1371/journal.pone.0041942)
Supplement: Table S1 — Characteristics of PCR Primer Sets and Products. (DOC) [file pone.0041942.s004.doc]

**Table S1. Characteristics of PCR Primer Sets and Products.**

| Gene | Size of Product | Primer Sequences (5’→3’) |
| --- | --- | --- |
| GAPDH | 200bp | Forward: ACCCAGAAGACTGTGGATGG  Reverse: TCTAGACGGCAGGTCAGGTC |
| E-Cadherin | 200bp | Forward: TGCCCAGAAAATGAAAAAGG  Reverse: GTGTATGTGGCAATGCGTTC |
| Vimentin | 163bp | Forward: GAGAACTTTGCCGTTGAAGC  Reverse: GCTTCCTGTAGGTGGCAATC |
| Snail1 | 234bp | Forward: CCTCCCTGTCAGATGAGGAC  Reverse: CCAGGCTGAGGTATTCCTTG |
| Snail 2 | 158bp | Forward: GGGGAGAAGCCTTTTTCTTG  Reverse: TCCTCATGTTTGTGCAGGAG |
| Twist | 201bp | Forward: GGAGTCCGCAGTCTTACGAG  Reverse: TCTGGAGGACCTGGTAGAGG |
| CD68 | 166bp | Forward: GACCCACGACTGCCACTC  Reverse: GTGCTGTTGCTTGTTGGATG |
| CD163 | 146bp | Forward: CGAGTTAACGCCAGTAAGG  Reverse: GAACATGTCACGCCAGC |
| CD204 | 366bp | Forward: CCAGGGACATGGGAATGCAA  Reverse: CCAGTGGGACCTCGATCTCC |
