# Supplementary material for: Tumor Associated Macrophage × Cancer Cell Hybrids May Acquire Cancer Stem Cell Properties in Breast Cancer
Source: PLoS One. 2012 Jul 25;7(7):e41942. doi: 10.1371/journal.pone.0041942 (PMC3405038; doi:10.1371/journal.pone.0041942)
Supplement: Table S2 — Tumorigenicity of the hybrids was enhanced in NOD/SCID mice. (DOC) [file pone.0041942.s005.doc]

Table S2. Tumorigenicity of the hybrids was enhanced in NOD/SCID mice.

|  | 1 | 2 | 3 | 4 | 5 | 6 | 7 | 8 | 9 | 10* |
| --- | --- | --- | --- | --- | --- | --- | --- | --- | --- | --- |
| MCF-7 | 0/3 | 0/3 | 0/3 | 0/3 | 0/3 | 0/3 | 0/3 | 0/3 | 0/3 | 0/3 |
| MCF-7 Fused | 0/3 | 1/3 | 2/3 | 3/3 | 3/3 | 3/3 | 3/3 | 3/3 | 3/3 | 3/3 |
| MDA-MB-231 | 0/3 | 0/3 | 1/3 | 3/3 | 3/3 | 3/3 | 3/3 | 3/3 | 3/3 | 3/3 |
| MDA-MB-231 Fused | 0/3 | 2/3 | 3/3 | 3/3 | 3/3 | 3/3 | 3/3 | 3/3 | 3/3 | 3/3 |

* Weeks after injection.
