# Supplementary figures and images for: Expression of Concern: Tumor Associated Macrophage × Cancer Cell Hybrids May Acquire Cancer Stem Cell Properties in Breast Cancer
Source: PLoS One. 2026 Feb 10;21(2):e0342526. doi: 10.1371/journal.pone.0342526 (PMC12890166; doi:10.1371/journal.pone.0342526)

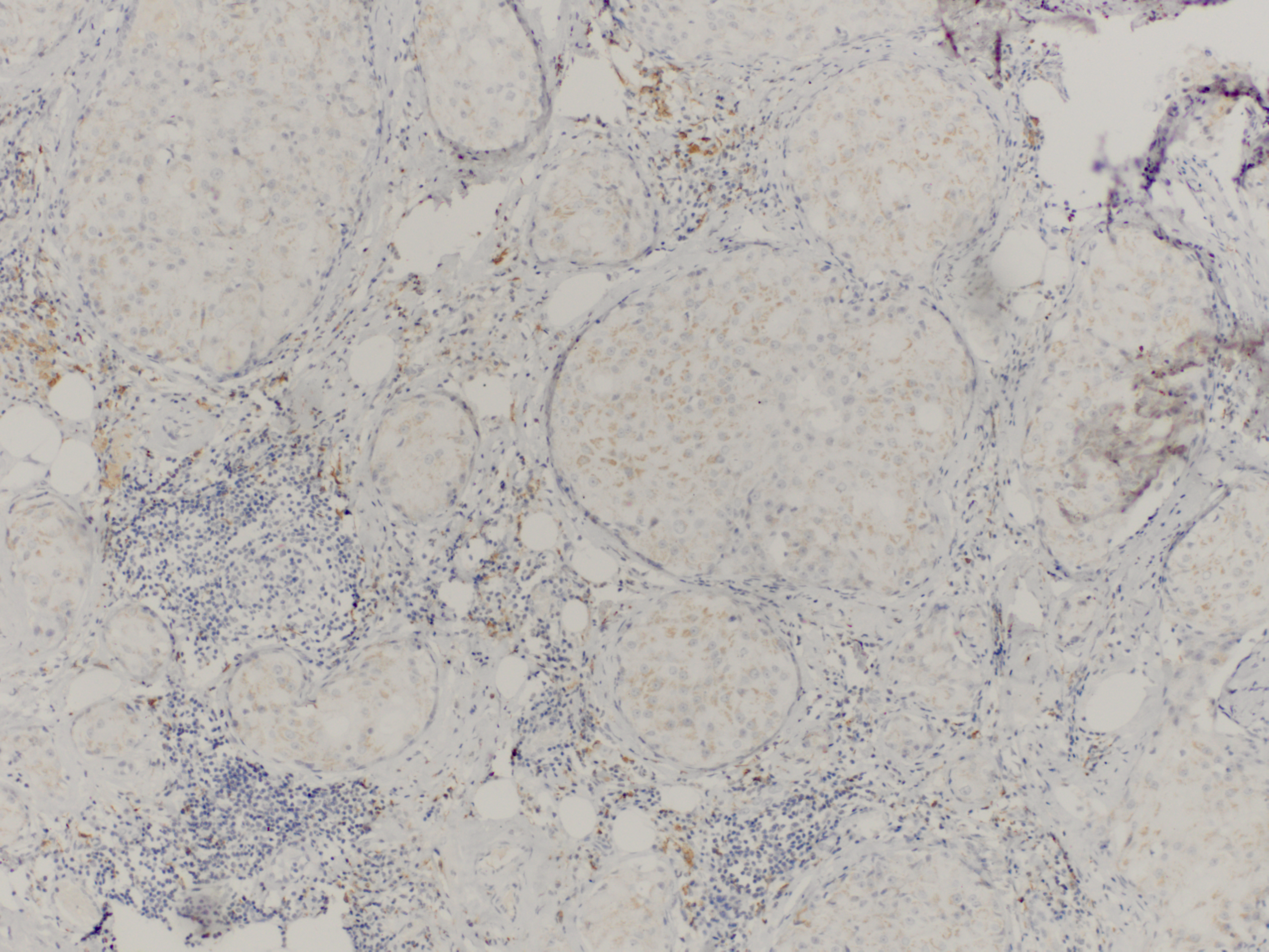

Supplement: S1 File — (ZIP) [file pone.0342526.s001.zip › S1 File/Figure 1A09-7217F4AER-DCIS.tif]

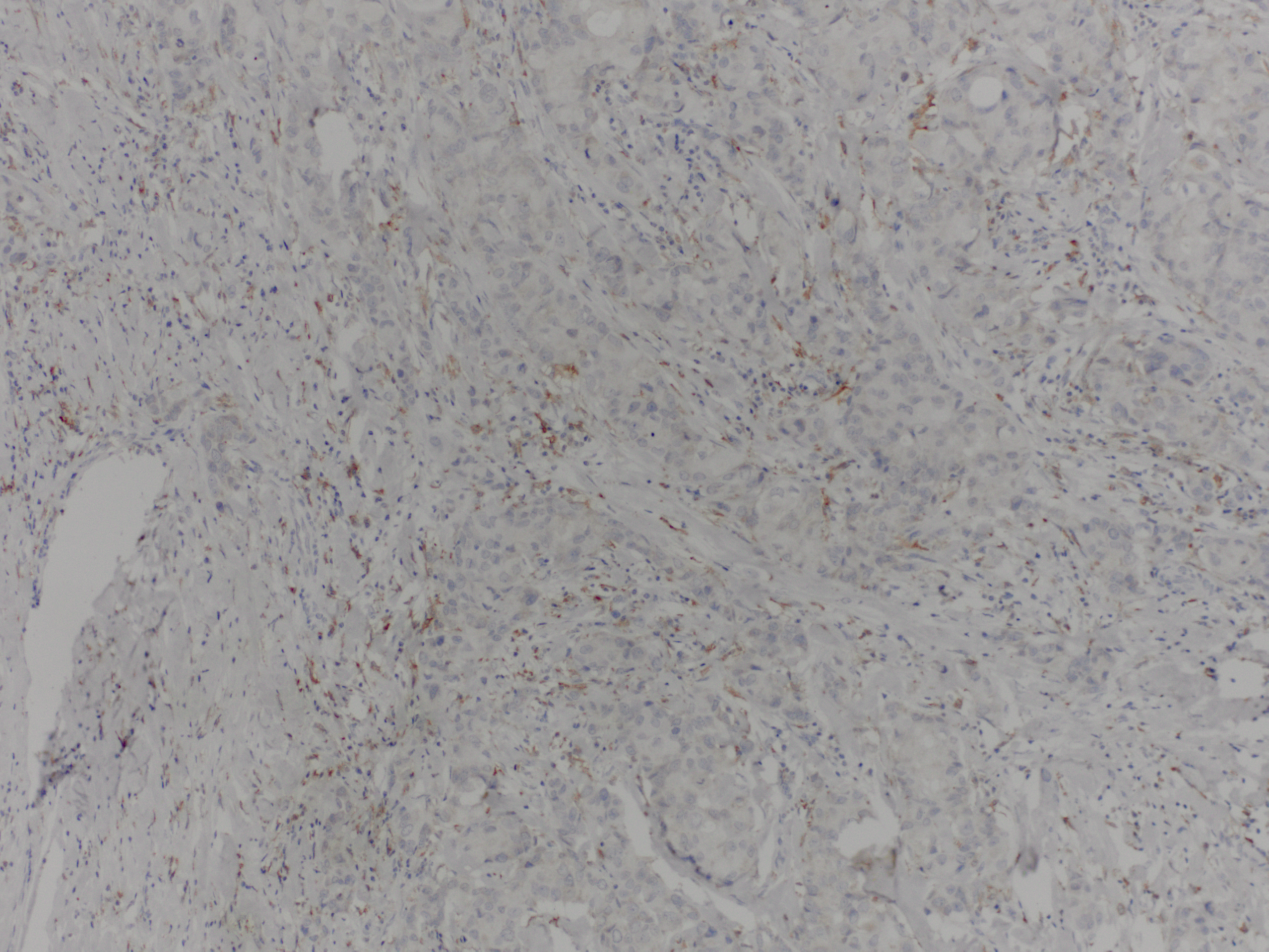

Supplement: S1 File — (ZIP) [file pone.0342526.s001.zip › S1 File/Figure 1B 09-6773P4E ER-IDC.tif]

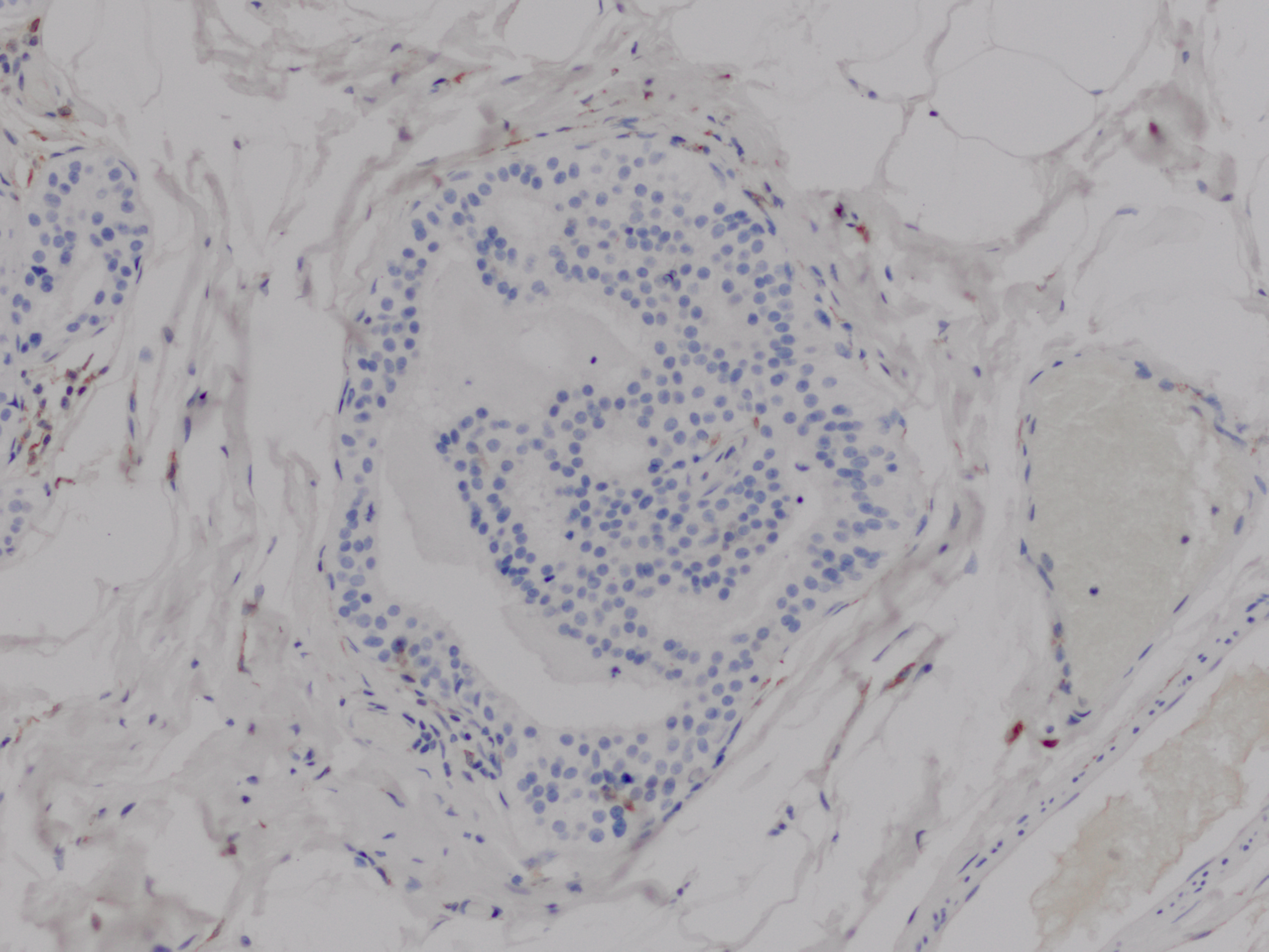

Supplement: S1 File — (ZIP) [file pone.0342526.s001.zip › S1 File/FIGURE 1C 08-11844B4DER+++DCIS.tif]

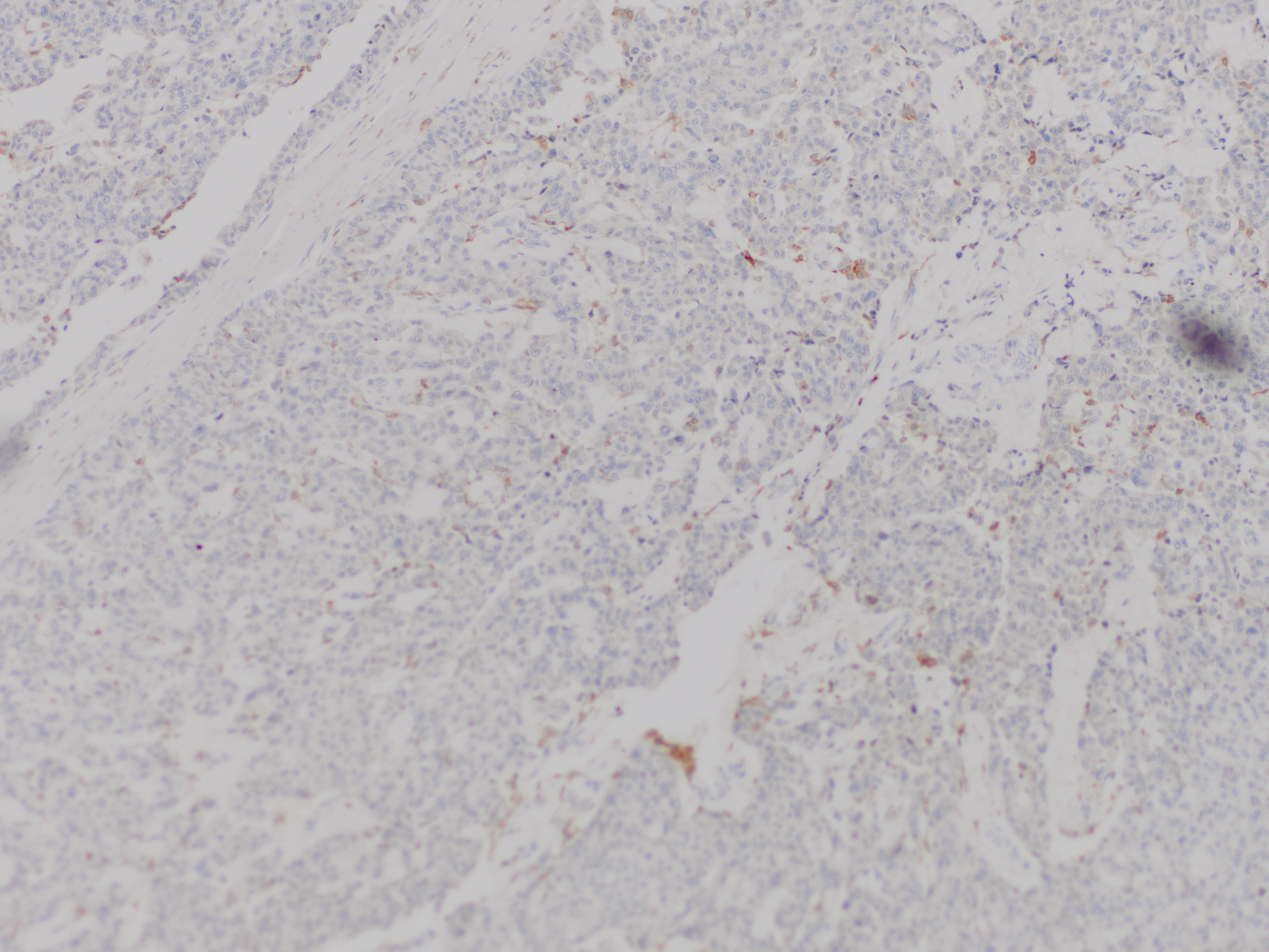

Supplement: S1 File — (ZIP) [file pone.0342526.s001.zip › S1 File/Figure 1D09-6238F4FER+++IDC.tif]

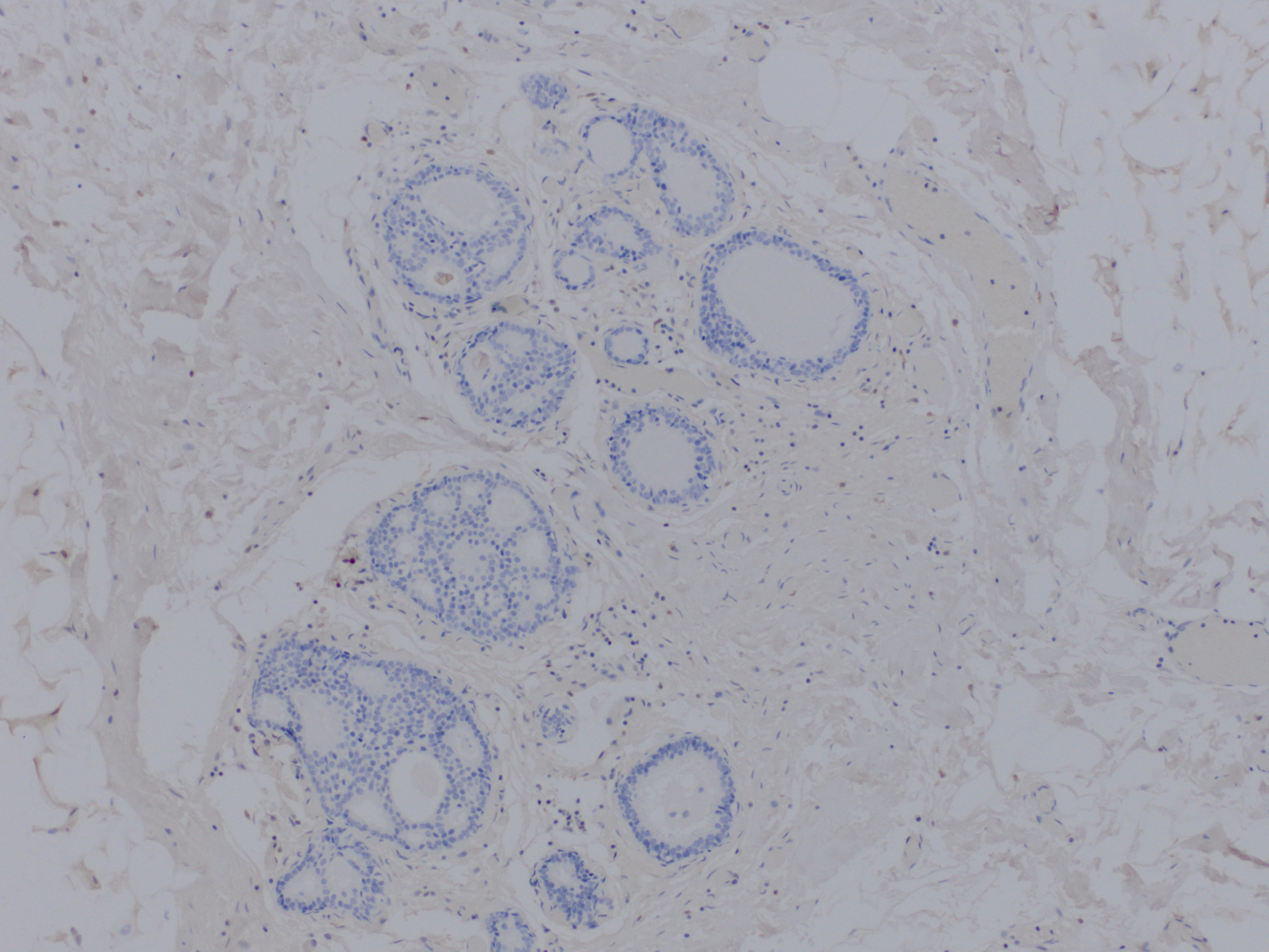

Supplement: S1 File — (ZIP) [file pone.0342526.s001.zip › S1 File/Fihure 1E 09-5952E4BNormal mammary duct.tif]

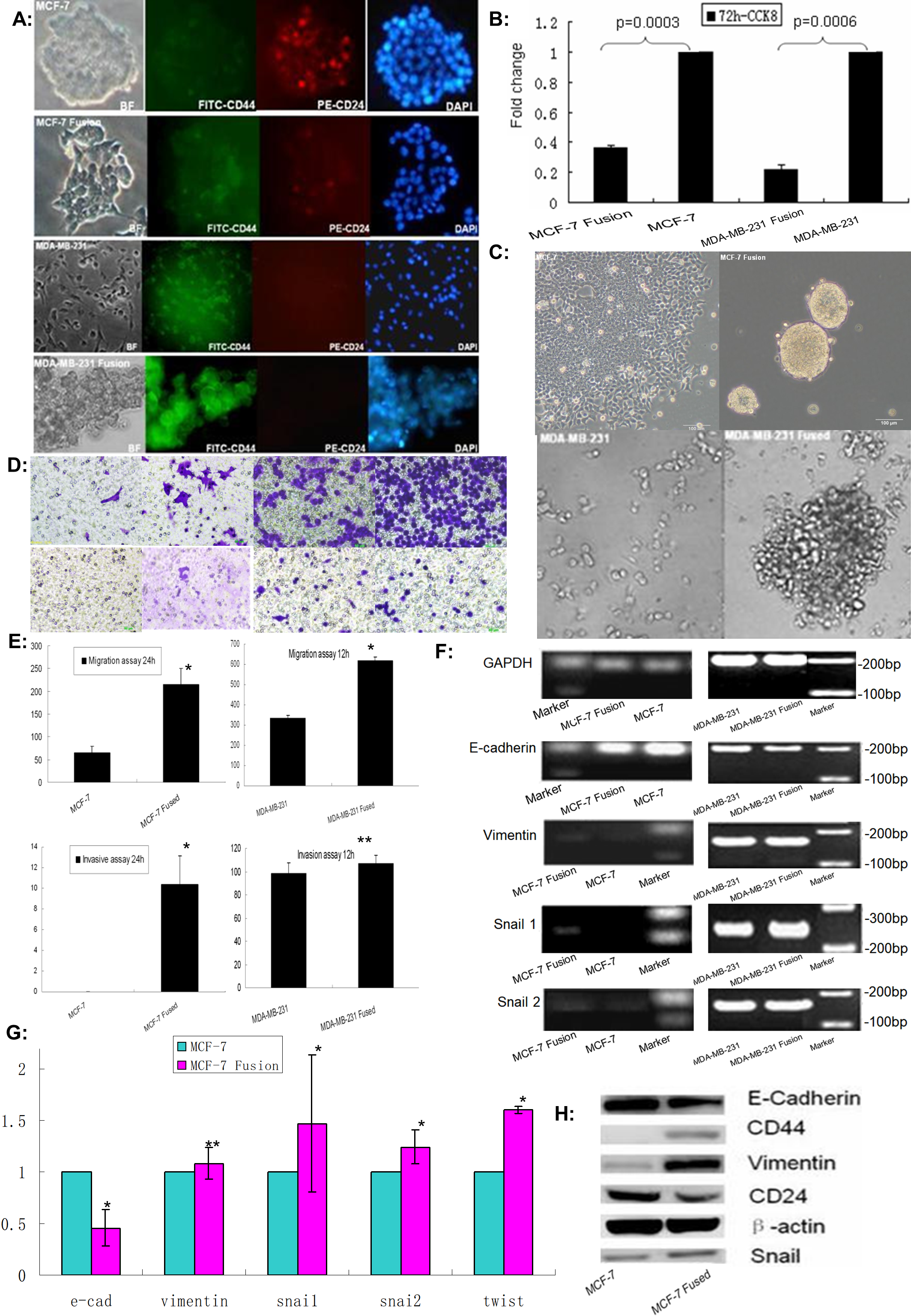

Supplement: S4 File — (ZIP) [file pone.0342526.s004.zip › S4 File/Figure 4.tif]
